# Telemedicine documentation in neurology and telestroke: a global scoping review

**DOI:** 10.1007/s10072-026-09165-3

**Published:** 2026-06-09

**Authors:** João Brainer Clares de Andrade, Claudia Galindo Novoa, Ivan Torres Pisa, Thiago S Carneiro, Nathalia Souza de Oliveira, Carlos Eduardo Rodriguez, George N Nunes Mendes

**Affiliations:** 1 Department of Neurology and Health Informatics, Federal University of Sao Paulo, Rua Botucatu, 862 Vila Clementino, Sao Paulo, CEP 04021-001 SP Brazil; 2 Academic Research Organization, Hospital Israelita Albert Einstein, Sao Paulo, Brazil; 3 Bioengineering Laborator, Institute of Aeronautics Technology, São José dos Campos, São Paulo, Brazil; 4 Department of Neurocritical Care, University of Florida, Gainesville, USA; 5 School of Medicine, University Center São Camilo, São Paulo, Brazil; 6 Department of Neurology, Centre Hospitalier de I’Universite de Montreal, Montreal, QC Canada

**Keywords:** Telemedicine, Documentation, Regulation, Neurology, Telestroke, Health Policy, Medical Informatics, Global Health

## Abstract

**Background:**

The rapid global expansion of telemedicine has created regulatory heterogeneity affecting documentation requirements, with particular implications for neurological services where documentation demands differ from general consultations.

**Objectives:**

To systematically examine documentation requirements for telemedicine across global jurisdictions, analyze convergence and divergence in regulatory approaches, evaluate specialty-specific requirements for neurology and telestroke, and explore liability frameworks for teleconsultations.

**Methods:**

Scoping review following PRISMA-ScR framework. Comprehensive searches of academic databases and grey literature through August 2025, examining sources published, enacted, or updated after January 1, 2020. A composite regulatory intensity score was developed to enable systematic comparison across jurisdictions.

**Results:**

Analysis of 147 regulatory sources across 52 jurisdictions revealed universal requirements for documentation elements while demonstrating substantial variation in consent documentation, data retention, electronic prescribing, and platform regulation. Regulatory intensity scores categorized jurisdictions across the full spectrum, with higher scores associated with more comprehensive documentation requirements.

**Conclusion:**

Telemedicine has transitioned from emergency contingency to regulated permanent care globally, with convergence on core principles but meaningful divergence in implementation specifics. Interoperability and mutual recognition rather than uniformity should guide regulatory harmonization efforts.

**Supplementary Information:**

The online version contains supplementary material available at 10.1007/s10072-026-09165-3.

## Introduction

The COVID-19 pandemic catalyzed unprecedented expansion of telemedicine services worldwide. From emergency utilization to permanent integration, health systems transitioned rapidly to accommodate remote care delivery, fundamentally reshaping healthcare documentation requirements [1]. Telemedicine, defined by the World Health Organization as the delivery of healthcare services using information and communication technologies to exchange valid information for diagnosis, treatment, and prevention of disease and injuries [2], has introduced novel challenges for maintaining comprehensive, legally defensible medical records that protect both patients and providers [3, 4].

Telemedicine documentation encompasses the systematic recording of synchronous or asynchronous clinical encounters conducted through telecommunications technology [5]. Requirements must address unique considerations including verification of patient identity, documentation of technology adequacy for clinical purposes, appropriate consent for remote care, and evidence of clinical decision-making quality comparable to in-person encounters [6]. The absence of physical examination creates documentation imperatives distinct from traditional consultations, requiring explicit recording of assessment limitations, alternative evaluation methods employed, and rationale for clinical decisions made under these constraints [7]. Documentation in telemedicine contexts specifically refers to the comprehensive recording of all clinical encounter elements as required by applicable laws, regulations, professional standards, and institutional policies, including elements unique to remote care delivery such as technology specifications, verification procedures, and acknowledgment of remote assessment limitations [8].

Regulatory heterogeneity across jurisdictions creates substantial compliance challenges for healthcare organizations operating multi-state or international telemedicine programs [9]. While some requirements demonstrate convergence—patient identification, consent documentation, medical record retention—implementation details vary significantly, creating complex compliance landscapes.

Neurological conditions present unique telemedicine documentation challenges. These challenges include: (1) visual observation limitations inherent to video-based neurological examination, where subtle findings such as fine tremor, mild ataxia, or sensory deficits may be difficult to assess remotely [10]; (2) narrow therapeutic windows in conditions such as acute stroke that require precise time-stamped documentation of clinical events and treatment decisions [11]; (3) standardized neurological assessment tools like the NIHSS requiring adaptation for remote administration with careful documentation of any modifications [12]; (4) patient-performed maneuvers during examination that necessitate real-time guidance and documentation of technique adequacy [13]; and (5) longitudinal tracking of disease progression in chronic neurological conditions requiring consistent documentation across virtual and in-person encounters [14]. Professional societies have developed specialty-specific guidelines often exceeding general regulatory requirements, creating additional compliance complexity [15].

Healthcare systems worldwide generally expect telemedicine encounters to maintain equivalent documentation standards to in-person consultations, though interpretation and implementation vary significantly [16]. These variations reflect differences in legal traditions, healthcare system structures, and digital infrastructure capabilities [17].

Liability frameworks in telemedicine refer to the legal structures that allocate professional responsibility and medicolegal accountability among healthcare providers participating in teleconsultation settings [18]. These frameworks are particularly critical for tele-interconsultations, where multiple physicians may be involved in patient care decisions across different geographic locations [19]. This scoping review systematically examines the global regulatory landscape of telemedicine documentation requirements, with particular focus on neurological services and telestroke care. We analyze convergences and divergences in regulatory approaches, examine specialty-specific requirements, and explore liability frameworks for teleconsultations.

## Methods

### Study design

This scoping review followed the Preferred Reporting Items for Systematic reviews and Meta-Analyses extension for Scoping Reviews (PRISMA-ScR) framework [20].

### Research questions

The review addressed four primary questions: (1) What are current regulatory requirements for telemedicine documentation across global jurisdictions? (2) How do documentation standards vary between general telemedicine and specialized neurology services, particularly telestroke? (3) What is the degree of convergence versus divergence in documentation requirements internationally? (4) How do liability frameworks for teleconsultations vary across jurisdictions?

### Search strategy

A comprehensive search strategy was developed with a medical librarian specializing in systematic reviews. Literature searches were conducted August 1–31, 2025, examining sources published, enacted, or updated after January 1, 2020; foundational regulatory documents enacted prior to January 2020 were included when they remained in force during the study period and provided essential baseline context for understanding current regulatory frameworks (e.g., China’s 2018 Internet Diagnosis and Treatment Measures and Indonesia’s 2019 telemedicine regulations, which remained authoritative during the study period). The January 2020 cutoff was selected because the COVID-19 pandemic represented a watershed moment for telemedicine regulation globally; pre-pandemic regulations were largely superseded by emergency measures and subsequent permanent frameworks, rendering earlier sources obsolete for contemporary practice [21, 22]. Academic databases included PubMed/MEDLINE, Scopus, Web of Science Core Collection, CINAHL Complete, and EMBASE. The strategy combined controlled vocabulary (MeSH terms: “Telemedicine,” “Telehealth,” “Remote Consultation,” “Documentation,” “Medical Records Systems, Computerized,” “Legal Liability,” “Government Regulation,” “Neurology,” “Stroke”) and free-text keywords organized into four concept blocks: telemedicine, regulation, documentation, and neurology. Boolean operators AND combined concept blocks, while OR linked synonyms within blocks.

Grey literature searches systematically examined: (1) National health authorities: Official websites of health ministries from WHO member states (*n* = 194); (2) Medical regulatory bodies from jurisdictions with established telemedicine programs (*n* = 78); (3) Professional societies: International and national neurology associations (*n* = 45), stroke organizations (*n* = 23), telemedicine associations (*n* = 31); (4) International organizations: WHO, OECD, Pan American Health Organization, European Commission; (5) Legal databases where accessible. Grey literature explicitly included ministerial health policies, national telemedicine strategies, official government regulations and decrees, and national digital health frameworks published by government agencies [23]. Search terms were translated into Spanish, Portuguese, French, German, and Mandarin for non-Anglophone jurisdictions, with native-speaking co-investigators verifying translations.

### Selection criteria and screening

Two independent reviewers (JBCA, GNNM) screened titles, abstracts, and full texts using Covidence systematic review software. Disagreements were resolved through discussion, with a third reviewer (TSC) available for arbitration. Inter-rater agreement for full-text screening achieved Cohen’s kappa of 0.87 (95% CI: 0.82–0.91), indicating strong concordance.

### Data extraction

Data extraction utilized a standardized form pilot-tested on 10 diverse sources. Extracted elements included: source characteristics, regulatory framework, general documentation requirements, specific requirements, technology specifications, electronic signatures and prescriptions, data retention and privacy, neurology-specific provisions, and enforcement mechanisms. Two reviewers (TSC, JBCA) independently extracted data from 20% of included sources (inter-rater agreement κ = 0.84), with discrepancies resolved through consensus.

### Regulatory intensity score methodology

To enable comparative analysis across jurisdictions with heterogeneous regulatory approaches, we developed a composite regulatory intensity score. Score development followed an iterative thematic analysis approach with elements of modified Delphi process. Three investigators (JBCA, ITP, GNNM) independently reviewed initial regulatory frameworks and proposed domain categories. Through three rounds of structured discussion, consensus was achieved on the eight domains described below [24]. Domain weighting was informed by existing digital health maturity assessment frameworks, including the Digital Health Profile and Maturity Assessment Toolkit co-developed with WHO regional offices, which emphasizes governance, workforce, and standards as foundational elements [25]. This multidimensional metric quantifies comprehensiveness and specificity of telemedicine documentation requirements across eight domains:

1. Core Documentation Elements (maximum 8 points): One point for each mandatory requirement among: patient identification with two-factor verification, provider credentials with license number, consultation start/end timestamps, relevant medical history, current medications and allergies, physical/virtual examination documentation, technology platform details, and structured follow-up arrangements.
2. Consent and Legal Frameworks (maximum 4 points): Scored hierarchically: implied consent (1 point), documented verbal consent (2 points), simple written consent (3 points), detailed written consent with technology limitations disclosure (4 points).
3. Technical Specifications (maximum 5 points): Points for mandatory video consultations, platform certification requirements, two-factor authentication mandates, biometric verification options, and EHR integration standards.
4. Specialty-Specific Requirements (maximum 5 points): Telestroke documentation protocols, mandatory NIHSS completion, time-stamped clinical event recording, standardized assessment scale requirements, and quality metrics reporting mandates.
5. Data Governance (maximum 3 points): Retention period requirements (1 point for 5–10 years; 2 points for > 10 years), explicit privacy law compliance specifications (0.5 points), and cross-border data transfer provisions (0.5 points).

Additional weighted elements: Platform licensing requirements (+ 3 points, reflecting systemic regulatory maturity—justified by correlation with comprehensive governance frameworks in Singapore, UAE, and Germany); retention periods exceeding 15 years (+ 1 point, indicating comprehensive medicolegal protection aligned with extended statute of limitations); evidence-based telestroke protocols aligned with AHA/ASA or ESO guidelines (+ 2 points, correlating with improved quality outcomes in stroke care).

Total possible score: 31 points (Domain subtotal: 8 + 4+5 + 5+3 = 25 points; Additional elements: 3 + 1+2 = 6 points). Scores were categorized as: Very High (> 26 points), High (21–26), Medium-High (16–20), Medium (11–15), Low-Medium (6–10), and Low (< 6). Two investigators (ITP, JCL) independently scored all jurisdictions (inter-rater agreement κ = 0.79); disagreements > 3 points were adjudicated through team discussion. Limitations of this scoring approach are acknowledged: weighting inherently reflects value judgments about regulatory maturity, and scores may favor civil law jurisdictions with prescriptive regulations over common law systems employing principles-based frameworks. External validation against patient safety metrics, malpractice claims data, and quality indicators is required before broader application [26, 27].

### Data synthesis

Quantitative synthesis employed descriptive statistics (frequencies, percentages, means, standard deviations, ranges) using R version 4.3.1. Qualitative synthesis used combined deductive and inductive thematic analysis following Braun and Clarke’s framework [28]. Deductive themes based on research questions included documentation requirements, consent procedures, technology requirements, and liability allocation. Inductive themes emerged through iterative review: platform regulation models, emergency-to-permanent provision transitions, equity considerations, and convergence-divergence patterns. Thematic coding was conducted independently by two reviewers (JBCA, CGN) using NVivo 14 software, with coded segments consolidated through consensus.

Complete jurisdiction-specific breakdowns and a step-by-step of the main analysis are available in Supplementary Material 1.

## Results

### Study selection

**Fig. 1:**
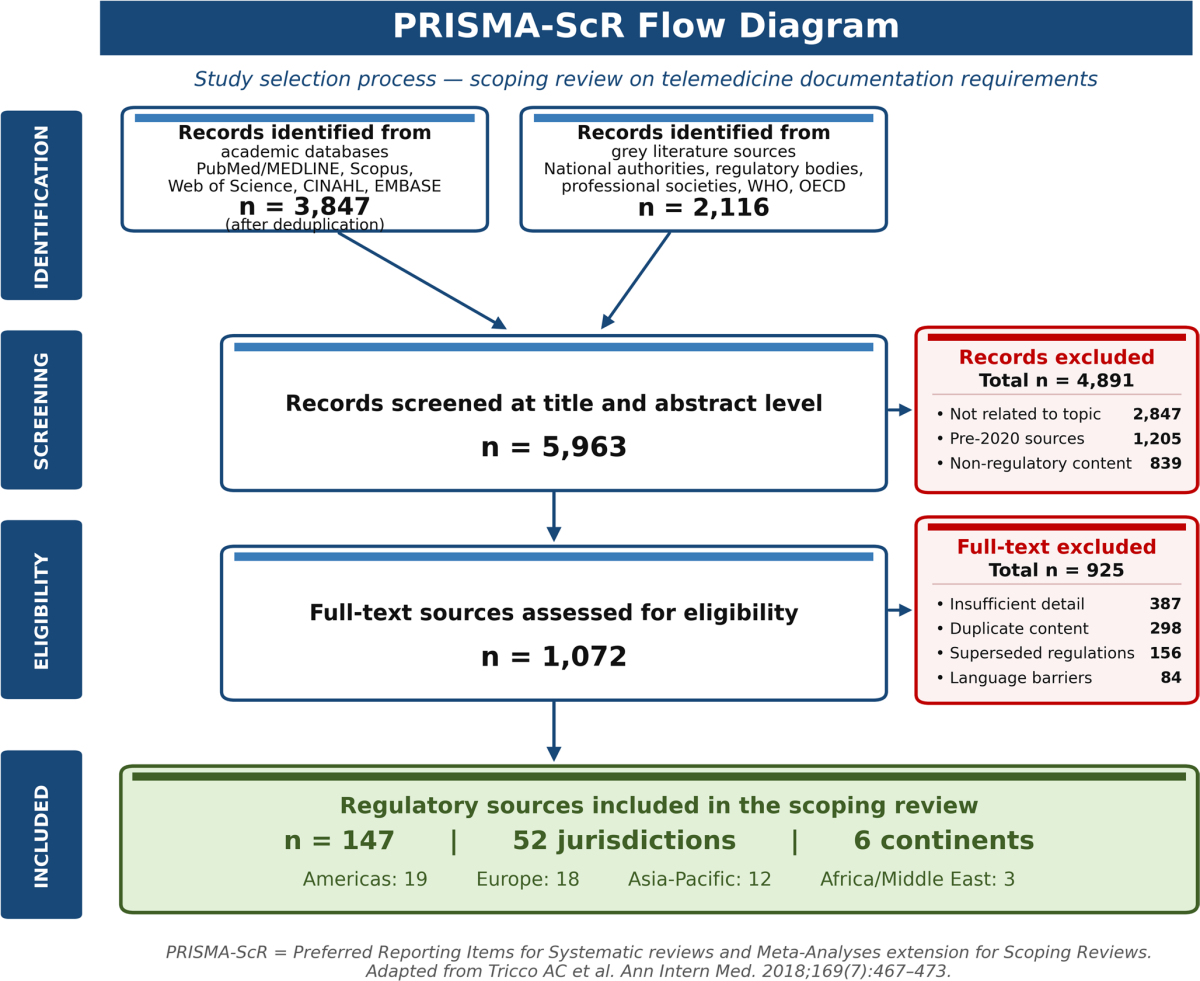
PRISMA-ScR flow diagram of the study selection process. Legend. PRISMA-ScR (Preferred Reporting Items for Systematic reviews and Meta-Analyses extension for Scoping Reviews) flow diagram illustrating the four-stage study selection process. A total of 3,847 unique records were identified through academic databases (PubMed/MEDLINE, Scopus, Web of Science Core Collection, CINAHL Complete, and EMBASE) after deduplication, and 2,116 sources were retrieved from grey literature, yielding 5,963 records screened at title and abstract level. After excluding 4,891 records (not related to telemedicine documentation: *n* = 2,847; pre-2020 sources: *n* = 1,205; non-regulatory content: *n* = 839), 1,072 sources were assessed for full-text eligibility. An additional 925 sources were excluded (insufficient documentation detail: *n* = 387; duplicate content: *n* = 298; superseded regulations: *n* = 156; language barriers: *n* = 84), resulting in 147 regulatory sources from 52 jurisdictions across six continents included in the qualitative synthesis (Americas: *n* = 19; Europe: *n* = 18; Asia-Pacific: *n* = 12; Africa/Middle East: *n* = 3)

The study selection process is summarized in Fig. 1 in accordance with the PRISMA-ScR framework. The comprehensive search yielded 3,847 unique records after deduplication from academic databases and 2,116 grey literature sources. Title and abstract screening excluded 4,891 records (not related to telemedicine documentation *n* = 2,847; pre-2020 sources *n* = 1,205; non-regulatory content *n* = 839). Full-text assessment of 1,072 sources excluded 925 (insufficient documentation detail *n* = 387; duplicate content *n* = 298; superseded regulations *n* = 156; language barriers *n* = 84). Finally, 147 regulatory sources from 52 jurisdictions across six continents were included. For clarification, these 52 jurisdictions comprise distinct regulatory units including sovereign nations (*n* = 47), sub-national states with independent healthcare licensing authority (*n* = 4, specifically US states with unique telemedicine frameworks), and distinct regulatory regions within countries (*n* = 1, Dubai/UAE with separate healthcare licensing from federal UAE regulations) [29].

**Fig. 2:**
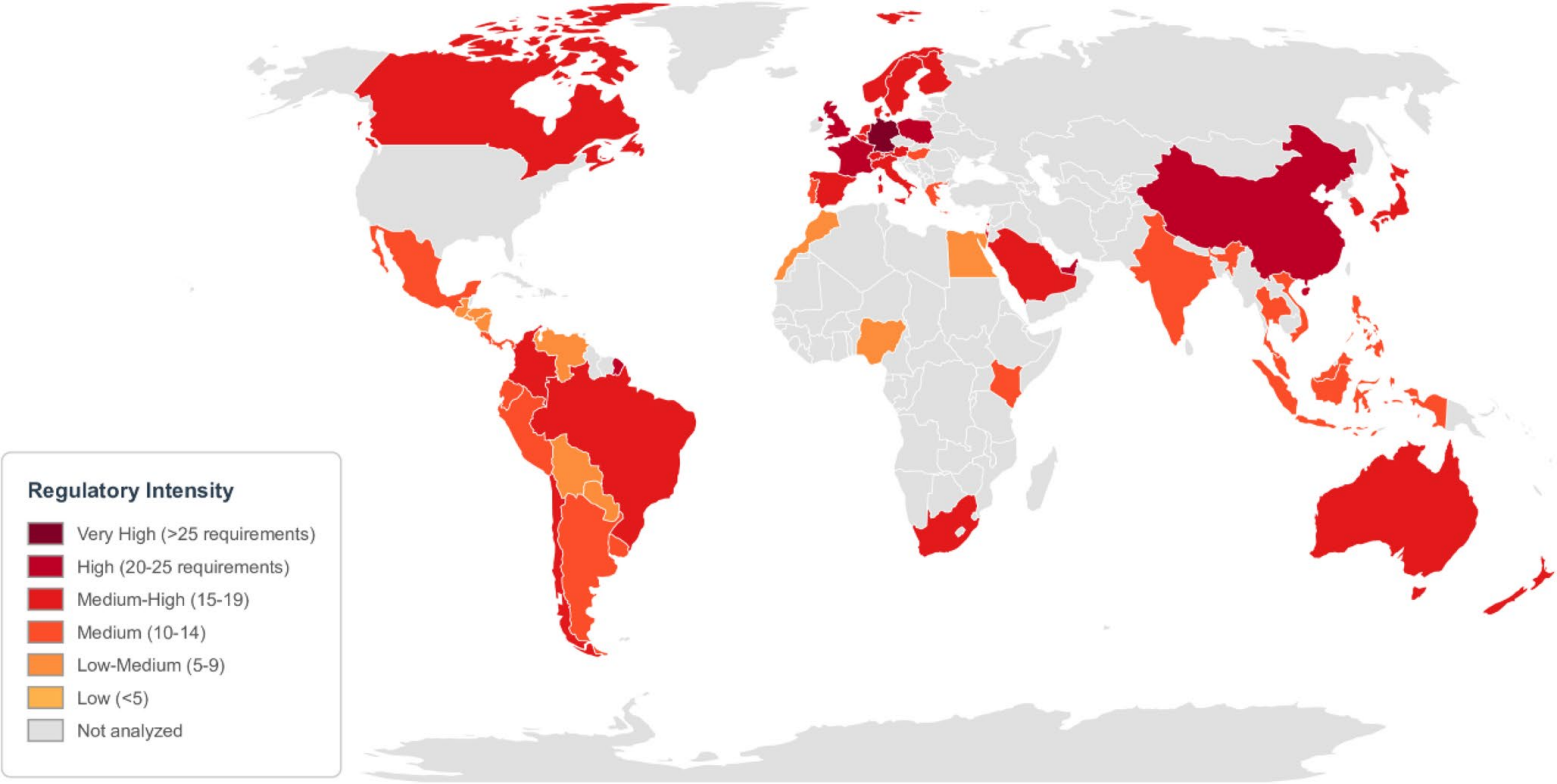
Global distribution of telemedicine documentation requirements across 52 jurisdictions (2020–2024). Legend. Heat map showing regulatory intensity scores derived from systematic analysis of 147 regulatory sources. The intensity score for each jurisdiction was calculated based on the cumulative presence of mandatory documentation requirements across multiple domains: (1) Core Documentation Elements (8 items): Patient identification, provider credentials, consultation timestamps, medical history, medications/allergies, physical/virtual examination documentation, technology platform details, and follow-up arrangements. (2) Consent and Legal Requirements (4 items): Type of consent required (implied, verbal, simple written, or detailed written), electronic signature frameworks, e-prescription capabilities, and controlled substance prescribing restrictions. (3) Technical Specifications (5 items): Video consultation mandates, platform certification requirements, two-factor authentication, biometric verification options, and EHR integration standards. (4) Specialty-Specific Requirements (5 items): Telestroke documentation standards, NIHSS completion requirements, time-stamped clinical events, standardized assessment scales, and quality metrics reporting. (5) Data Governance (3 items): Retention period requirements (weighted by duration), privacy law compliance specifications, and cross-border data transfer provisions. Each requirement was assigned 1 point if mandatory, 0.5 points if recommended/optional, resulting in a maximum possible score of 25 points. Additional weighting was applied for: (1) platform licensing requirements (+ 3 points), reflecting systemic regulatory approach; (2) retention periods exceeding 15 years (+ 1 point); and (3) comprehensive telestroke protocols (+ 2 points), given their correlation with quality outcomes. Color gradients represent regulatory intensity: Very High (> 25 requirements, dark red), High (20–25, red), Medium-High (15–19, orange-red), Medium (10–14, orange), Low-Medium (5–9, light orange), Low (< 5, pale yellow), and Not Analyzed (gray). Data sources considered national legislation

Sources comprised national legislation (*n* = 63, 42.9%), sub-national/state regulations (*n* = 28, 19.0%), professional regulatory body guidance (*n* = 31, 21.1%), government policy documents (*n* = 15, 10.2%), and peer-reviewed regulatory analyses (*n* = 10, 6.8%). Geographic distribution showed Americas (*n* = 19 jurisdictions, 36.5%), Europe (*n* = 18, 34.6%), Asia-Pacific (*n* = 12, 23.1%), and Africa/Middle East (*n* = 3, 5.8%). Figure 2 illustrates the regulatory intensity scores derived from systematic analysis of the 147 regulatory sources across all jurisdictions.

### Universal documentation requirements (All conditions)

The following requirements apply to all telemedicine encounters regardless of specialty. Healthcare systems worldwide require telemedicine encounters to meet equivalent documentation standards as in-person care, though implementation varies [16]. Universal documentation elements include patient identification, provider identification, date/time of consultation, chief complaint, assessment/diagnosis, treatment plan, and consent documentation.

Table 1 presents minimum documentation requirements for general telemedicine consultations, demonstrating convergence on core elements but divergence in specificity. Consent documentation requirements show significant variation: some jurisdictions accept implied consent, others require documented verbal consent, many mandate simple written consent, and some require detailed written consent with specific disclosures about technology limitations, data security risks, and remote assessment constraints [5].

**Table 1:** Universal and variable documentation requirements across jurisdictions

Documentation element	Required by *n* (%)	Specification details	Regional variations
Patient identification	52 (100%)	Two-factor verification in 98%	Asia-Pacific: biometric options (42%)
Provider credentials	52 (100%)	License number mandatory	Europe: specialty registration (78%)
Consultation timestamps	52 (100%)	Start/end times in 67%	Americas: duration tracking (84%)
Medical history	51 (98.1%)	Relevant past history	Africa/ME: HIV status (63%)
Medications/allergies	51 (98.1%)	Current list required	Europe: e-prescription link (89%)
Physical/virtual exam	50 (96.2%)	Technology limitations noted	Asia: mandatory video (75%)
Technology platform	43 (82.7%)	Platform name/version	ME: platform license # (88%)
Follow-up arrangements	49 (94.2%)	Next appointment/contingencies	Americas: referral tracking (71%)

**Legend**. Distribution of telemedicine documentation requirements across 52 global jurisdictions, showing percentage compliance and regional variations. HPI = History of Present Illness. Two-factor verification was nearly universal (98%), with Asia-Pacific regions leading in biometric authentication adoption (42%). While core elements achieved 100% compliance, technology platform documentation showed the greatest variability (82.7%), reflecting differences in regulatory maturity. The Middle East (ME) demonstrated the highest platform documentation requirements (88%), correlating with their facility-level licensing models. Data compiled from national regulatory frameworks and professional guidelines

### Data retention periods

Data retention periods demonstrate striking heterogeneity. Table 2 illustrates retention period variations between jurisdictions, with India mandating 3-year retention for physician-maintained inpatient records [30], though many hospitals retain records for 10 + years. Indonesia requires 25-year retention for electronic medical records under the Minister of Health Regulation (Permenkes) No. 24 of 2022 on Medical Records, with telemedicine encounters subject to identical retention requirements as in-person care [31]. Retention period variation across European countries—with some jurisdictions requiring up to 30 years for specific record types—derives from national medical record and medicolegal statutes rather than from the GDPR, which establishes principles of storage limitation and data minimization without prescribing specific retention durations [32, 33]. For example, German medical record law (Berufsordnung für Ärzte) requires 10-year minimum retention, while French Code de la Santé Publique mandates 20 years for certain records [34].

**Table 2:** Medical record retention requirements by region

Retention period	Jurisdictions (*n*, %)	Primary rationale	Representative countries
3–5 years	8 (15.4%)	Malpractice statute alignment	India, Thailand, Japan
6–7 years	11 (21.2%)	Standard medical records	Australia, UK, Canada
8–10 years	14 (26.9%)	Extended liability protection	Germany, USA, Italy
11–15 years	9 (17.3%)	Long-term continuity	China, Colombia, Chile
16–20 years	7 (13.5%)	Lifetime documentation	France, Poland, Brazil
> 20 years	3 (5.7%)	Permanent health record	Kenya, Indonesia, Vietnam

**Legend**. Mandated retention periods for telemedicine records across 52 jurisdictions, stratified by duration and primary rationale. SD = Standard Deviation. Mean retention was 11.3 years (SD ± 6.2). The fourfold difference between shortest (India, 3 years) and longest (Indonesia, 25 years) requirements reflects divergent legal philosophies regarding statute of limitations versus lifetime health documentation. European countries showed the highest mean retention (13.8 years) compared to Asia-Pacific (9.1 years), suggesting correlation with data protection regulations like GDPR. Data sourced from national health regulations and medical board guidelines

### Neurology-specific documentation standards (Neurological conditions only)

In contrast to the general requirements described above, the following documentation standards apply specifically to neurological telemedicine encounters. Analysis of neurology-specific requirements revealed more detailed standards than general telemedicine regulations, though the source of these requirements varied significantly across jurisdictions. We classified requirements according to their regulatory authority: (1) binding legislation or government regulation; (2) professional regulatory body requirements (e.g., medical council or licensing board mandates); (3) clinical practice guidelines from professional societies (e.g., AHA/ASA, ESO); and (4) accreditation or quality program standards (e.g., stroke center certification requirements). The following percentages reflect aggregated requirements across all four categories, as jurisdictions variably incorporate these elements through different regulatory mechanisms:

Among 52 jurisdictions, time-stamped documentation for acute stroke teleconsultations was required by binding regulation in 23 jurisdictions (44.2%), by professional regulatory body mandates in an additional 14 jurisdictions (26.9%), and through clinical guidelines or accreditation standards in the remaining 12 jurisdictions with any telestroke requirements (23.1%); aggregated across all regulatory mechanisms, 49 jurisdictions (94.2%) incorporated time-stamping requirements. Complete NIHSS score documentation followed a similar pattern: binding regulation in 18 jurisdictions (34.6%), professional body requirements in 12 jurisdictions (23.1%), and guidelines/accreditation in 10 jurisdictions (19.2%), for an aggregate of 40 jurisdictions (76.9%). All 52 jurisdictions (100%) require documentation of contraindications before thrombolytic therapy, though 31 (59.6%) establish this through clinical guidelines rather than binding law. Risk-benefit discussion documentation is incorporated in 42 jurisdictions (80.8%) across regulatory mechanisms, and consent provider identification in 37 jurisdictions (71.2%). A detailed matrix mapping each jurisdiction to specific regulatory sources for each requirement is provided in **Supplementary Material ****2**,** Table ****S1**.

The 2019 AHA/ASA guidelines recommend telestroke as a beneficial intervention [15]. The 2017 American Telemedicine Association Telestroke Guidelines specify time-stamping requirements [11]. ESO’s 2018 recommendations address organizational standards [35]. Table 3 presents required elements across major guidelines for telestroke documentation.

**Table 3:** Telestroke documentation requirements - international comparison

Documentation element	AHA/ASA	ESO	WSO	National requirement (% countries)
Last known well time	Required	Required	Recommended	89%
Door/arrival time	Required	Required	Recommended	87%
Imaging completion time	Required	Required	Recommended	83%
Consultation start time	Required	Required	Recommended	91%
tPA decision time	Required	Required	Recommended	78%
Needle time (if given)	Required	Required	Required	94%
Complete NIHSS score	Required	Required	Recommended	76%
NIHSS components	Recommended	Required	Optional	43%
Contraindications assessed	Required	Required	Required	100%
Risk-benefit discussion	Required	Required	Recommended	81%
Consent provider identified	Required	Recommended	Recommended	72%

**Legend.** Comparison of telestroke documentation standards across three major international guidelines and national implementation rates among the 52 analyzed jurisdictions. AHA/ASA = American Heart Association/American Stroke Association; ESO = European Stroke Organisation; WSO = World Stroke Organization; NIHSS = National Institutes of Health Stroke Scale; tPA = tissue plasminogen activator

National requirement percentages reflect mandatory documentation elements in jurisdiction-specific telestroke protocols among all 52 jurisdictions analyzed (*n* = 52). Time-critical elements showed highest compliance (needle time 94.2%, *n* = 49/52), while detailed NIHSS component documentation remained variable (42.3%, *n* = 22/52). The universal requirement for contraindication assessment (100%, *n* = 52/52) reflects medicolegal imperatives in thrombolytic therapy decisions across all regulatory frameworks. Based on analysis of professional society guidelines [8, 10, 18] and national telestroke program requirements identified in the 147 regulatory sources

Documentation requirements for chronic neurological conditions show greater heterogeneity across jurisdictions (**Supplementary Material ****2**,** Table ****S2**). Requirements for chronic conditions varied substantially, with dementia documentation being most standardized (93% of jurisdictions required cognitive assessment scales) and headache disorders least standardized (12% required caregiver assessment documentation) [14].

### Electronic signatures and prescriptions

Electronic signature frameworks show convergence in many jurisdictions recognizing legal validity. Simple electronic signatures suffice for routine documentation in many jurisdictions, while some require advanced electronic signatures for prescriptions and consent [36].

E-prescription implementation varies considerably. Germany implemented mandatory e-prescriptions for statutory health insurance patients January 1, 2024 [37]. Poland’s system became obligatory January 8, 2020 [38]. Controlled substance e-prescribing remains restricted in most jurisdictions [39].

### Platform regulation models

Regulatory approaches to technology platforms reveal fundamental differences (**Supplementary Material ****2**,** Table ****S3**). Singapore implemented telemedicine platform licensing under its Healthcare Services Act starting mid-2022 [40]. Other jurisdictions regulate through provider-based frameworks [41].

### Teleconsultation liability frameworks

**Fig. 3:**
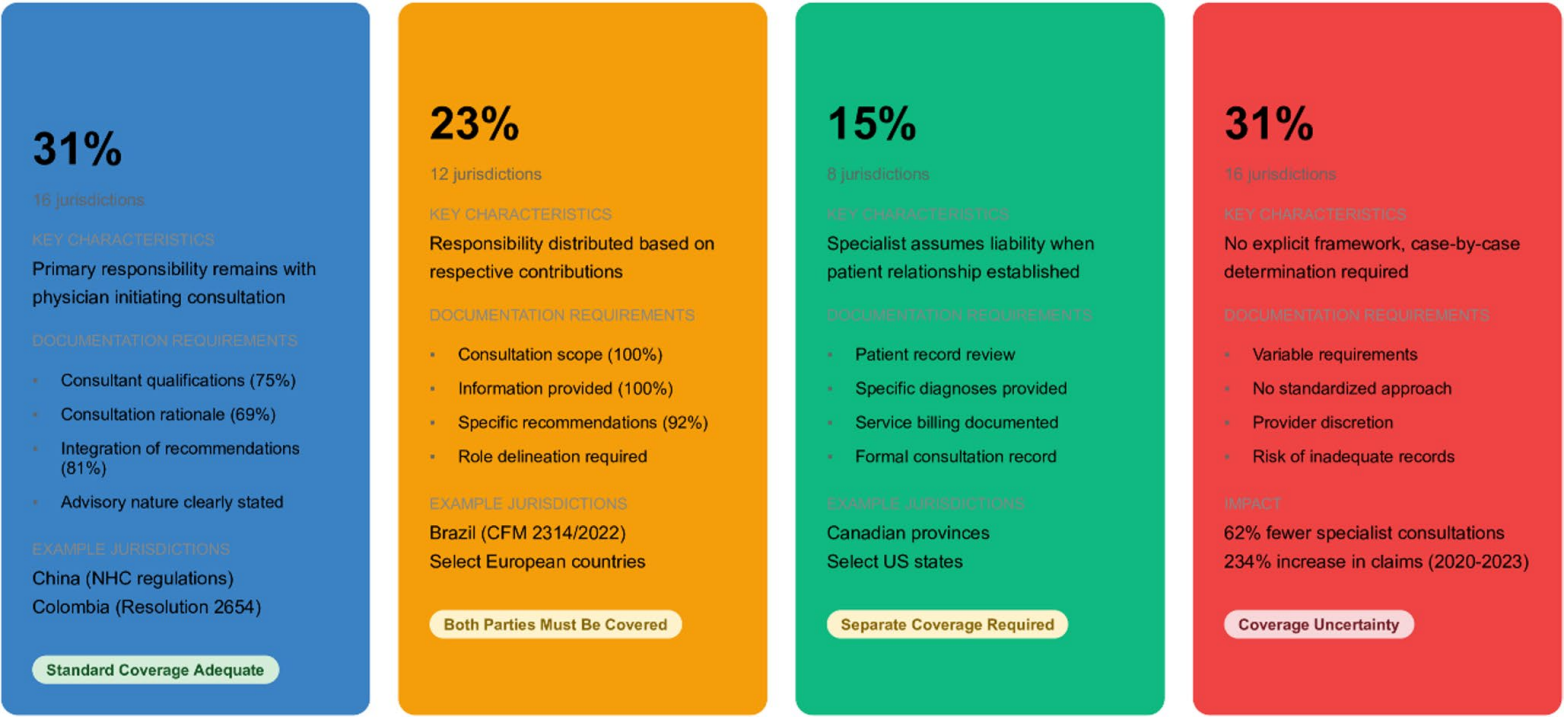
Tele-interconsultation liability models: distribution and characteristics across 52 jurisdictions. Legend. Matrix visualization displaying the four identified liability frameworks for physician-to-physician teleconsultations (tele-expertise), their prevalence, documentation requirements, and insurance implications. Data Structure: The visualization presents a horizontal stacked bar showing the proportional distribution of jurisdictions across four models, followed by detailed characteristic cards for each framework. Requesting Physician Primary (30.8%, *n* = 16): Assigns primary responsibility to the physician initiating the consultation. Documentation focuses on establishing the advisory nature of specialist input, with 12 of 16 jurisdictions (75%) requiring consultant qualification documentation, 11 (68.8%) mandating consultation rationale documentation, and 13 (81.3%) requiring documentation of recommendation integration. Examples include China (NHC Internet Hospital regulations, 2018) [27] and Colombia (Resolution 2654 of 2019). Shared Liability (23.1%, *n* = 12): Distributes responsibility based on respective physician contributions. Shows the most comprehensive documentation requirements with all 12 jurisdictions (100%) requiring consultation scope documentation and information provided, and 11 (91.7%) requiring specific recommendation documentation. Brazil’s CFM Resolution 2314/2022 exemplifies this approach [28]. Consultant Liability (15.4%, *n* = 8): Assigns responsibility to the remote specialist when a physician-patient relationship is established. Documentation centers on evidence of patient interaction, including all 8 jurisdictions (100%) requiring chart review documentation, 7 (87.5%) requiring specific diagnosis documentation, and 6 (75%) requiring documentation when services are billed directly. Canadian provincial regulations predominantly follow this model [28]. Undefined/Ambiguous (30.8%, *n* = 16): Lacks explicit regulatory framework, resulting in variable documentation practices and case-by-case liability determination. Professional association surveys from 8 of these jurisdictions identified medicolegal uncertainty as a barrier to teleconsultation adoption [29, 31]. Insurance coverage analysis revealed that only 38% of jurisdictions (*n* = 20 of 52 total) explicitly address tele-interconsultation in professional liability policies [31]. Key Findings: The equal distribution between requesting physician primary (30.8%) and undefined frameworks (30.8%) reveals a polarized regulatory landscape. Documentation requirements vary significantly across models, with shared liability frameworks demonstrating the highest specificity. The absence of explicit insurance coverage in 62% of jurisdictions creates potential gaps in professional protection for teleconsultants

Analysis of teleconsultation liability reveals significant variation. Figure 3 demonstrates the distribution of liability models across jurisdictions. China’s regulations explicitly state requesting medical institutions bear ultimate responsibility for diagnosis and treatment decisions [42]. Other countries establish shared liability models or maintain consultant liability when formal physician-patient relationships are established [43].

Many jurisdictions lack explicit frameworks, creating uncertainty [44]. Table 4 presents the distribution of liability frameworks. Among 16 jurisdictions (30.8%) with undefined or ambiguous tele-interconsultation liability frameworks, regulatory documentation and professional association reports revealed reduced adoption of specialist teleconsultation services. Survey data from professional medical associations in 8 of these jurisdictions indicated medicolegal exposure concerns as primary barriers to participation [44, 45].

**Table 4:** Tele-interconsultation liability models by jurisdiction

Liability model	Jurisdictions *n* (%)	Key determinants	Documentation requirements	Insurance implications
Requesting physician primary	16 (31%)	Final decision authority	Consultant input noted as advisory	Standard coverage
Shared liability	12 (23%)	Contribution level	Detailed role delineation	Both parties covered
Consultant liability	8 (15%)	Patient relationship establishment	Formal consultation documentation	Separate coverage needed
Undefined/ambiguous	16 (31%)	Case-by-case determination	Variable	Coverage uncertainty

**Legend.** Distribution of medicolegal liability frameworks for physician-to-physician teleconsultations (tele-expertise) across 52 jurisdictions. Nearly one-third of jurisdictions (31%) lack explicit frameworks, creating legal uncertainty for specialist consultations. Shared liability models (23%) showed the most comprehensive documentation requirements, mandating detailed role delineation in 100% of cases. Insurance coverage gaps were identified in 62% of jurisdictions, with only 38% explicitly addressing tele-interconsultation in professional liability policies. Analysis based on review of national medical liability frameworks and professional association guidelines

Jurisdictions with explicit liability frameworks (*n* = 36, 69.2%) demonstrated more comprehensive documentation requirements. Among these, 27 (75%) require explicit documentation of consultant qualifications, 25 (69.4%) mandate documentation of consultation rationale, and 29 (80.6%) require documentation of how specialist recommendations were integrated into patient care [18].

Professional liability insurance coverage for teleconsultations remains inconsistent, with survey data from medical liability insurers in 23 jurisdictions revealing only 14 (60.9%) explicitly include tele-interconsultation in standard policies [45]. Cross-border teleconsultations face additional coverage gaps, with 19 of 23 surveyed insurers (82.6%) excluding international consultations.

### Implementation challenges

Analysis identified systematic barriers to documentation compliance (**Supplementary Material ****2**,** Table ****S4**). Cross-jurisdictional licensing emerged as a primary barrier, followed by technology infrastructure limitations, provider training gaps, and EHR integration challenges [46]. Implementation facilitators associated with successful adoption included standardized EHR templates, mandatory training programs, and professional society engagement [47].

## Discussion

This comprehensive scoping review reveals a global healthcare system transitioning from emergency pandemic measures toward permanent telemedicine integration. While healthcare systems expect telemedicine to meet equivalent documentation standards as in-person care, implementation heterogeneity creates significant challenges for practitioners, organizations, and patients navigating cross-border care [16].

### Regulatory convergence and divergence

**Fig. 4:**
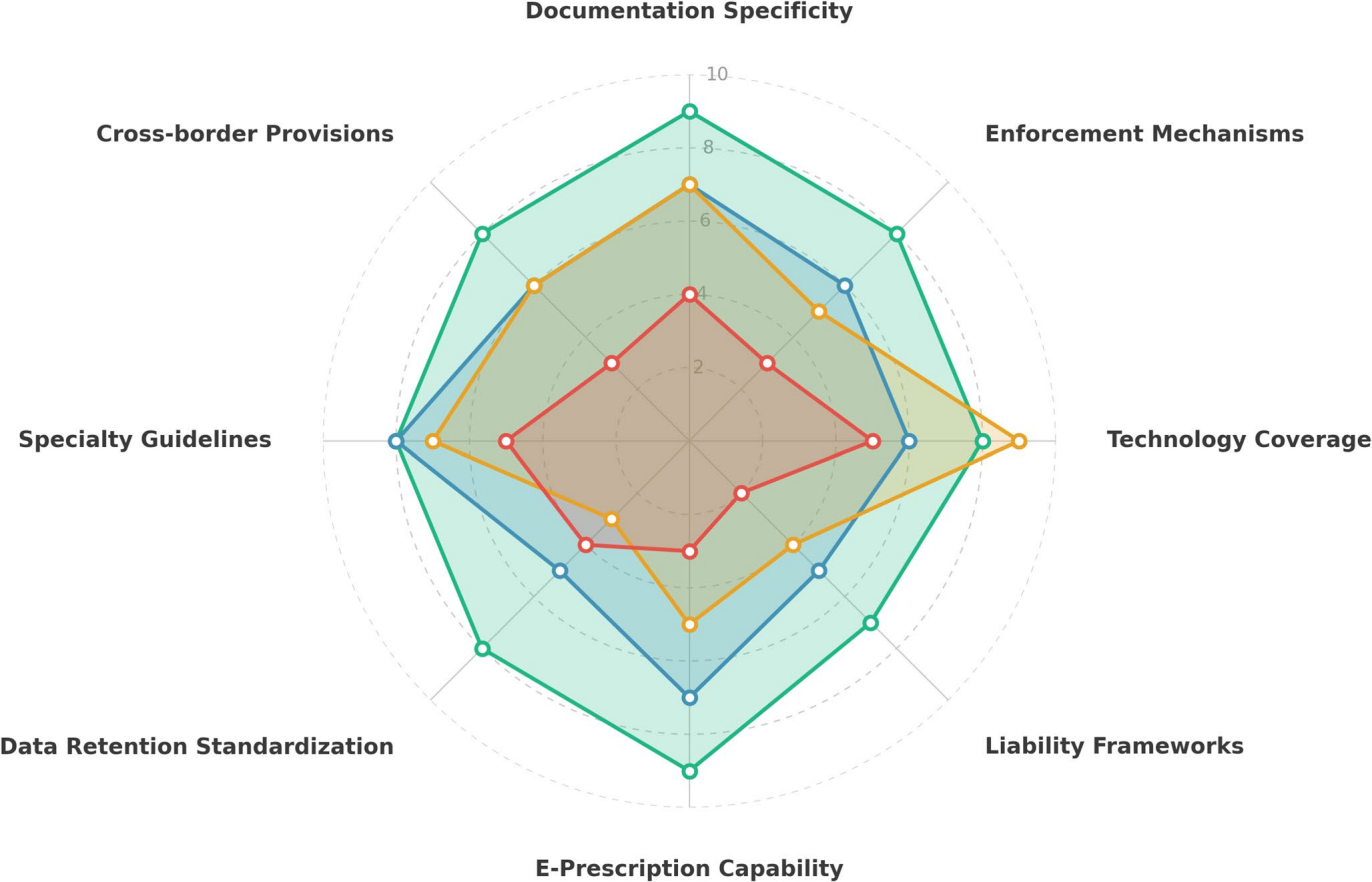
Telemedicine regulatory maturity assessment across global regions. Legend. Eight-dimensional radar chart comparing regulatory maturity across four geographic regions comprising 52 jurisdictions: Americas (in Blue, *n* = 19), Europe (in Green, *n* = 18), Asia-Pacific (in Orange, *n* = 12), and Africa/Middle East (in Red, *n* = 3). Maturity Index calculation considered each dimension was scored on a 0–10 scale based on quantitative analysis of 147 regulatory sources. The composite maturity index was calculated as follows: (1) Documentation Specificity (weight: 15%): Number of mandatory documentation elements divided by 30 (maximum identified), multiplied by 10. Europe scored 9/10 with 27 required elements versus Africa/ME at 4/10 with 12 elements. (2) Enforcement Mechanisms (weight: 15%): Composite score of: audit frequency (0–3 points), penalty severity (0–3 points), compliance monitoring systems (0–2 points), and regulatory body powers (0–2 points). (3) Technology Coverage (weight: 15%): Platform licensing (0–4 points) + EHR integration requirements (0–3 points) + technical certification mandates (0–3 points). Asia-Pacific scored highest (9/10) due to Singapore’s pioneering platform model adopted by 8 jurisdictions. (4) Liability Frameworks (weight: 15%): Jurisdictions with explicit frameworks scored 8–10; shared liability models 6–7; requesting physician primary 4–5; undefined frameworks 0–3. Europe’s 23% shared liability adoption yielded 7/10. (5) E-Prescription Capability (weight: 10%): Full implementation with controlled substances (8–10 points); integrated systems (5–7 points); pilot programs (2–4 points); absent (0–1 points). (6) Data Retention Standardization (weight: 10%): Inversely scored based on coefficient of variation. Europe’s consistency (SD ± 2.3 years) scored 8/10 versus Asia-Pacific’s extreme variation (3–25 years) scoring 3/10. (7) Specialty Guidelines (weight: 10%): Percentage of jurisdictions with telestroke requirements multiplied by comprehensiveness score (NIHSS documentation, time stamps, contraindication assessment). (8) Cross-border Provisions (weight: 10%): Mutual recognition agreements (0–4 points) + licensure portability (0–3 points) + international practice frameworks (0–3 points). Overall Maturity Score Calculation was the sum of (dimension score × weight) = Total percentage. Europe: 82% (strongest in documentation, enforcement, e-prescriptions). Americas: 68% (leading in specialty guidelines through AHA/ASA). Asia-Pacific: 65% (technology pioneers, inconsistent other dimensions). Africa/Middle East: 48% (emerging frameworks, significant gaps)

Convergence on core documentation elements demonstrates shared understanding of essential medical record components. However, divergence in specificity reflects fundamental differences in regulatory philosophy. Common law jurisdictions typically favor principle-based approaches allowing professional judgment, while civil law countries provide more prescriptive requirements [48]. This variation creates compliance complexity for international telemedicine providers navigating different requirements and fundamentally different regulatory approaches. Figure 4 shows regional maturity variations across key regulatory dimensions.

The striking variation in data retention periods—from 3 years in India to 25 years in Indonesia—has profound implications for storage costs, system design, and longitudinal care. These retention disparities reflect broader medical record governance frameworks rather than telemedicine-specific provisions. India’s NMC 2023 regulations establish a 3-year minimum retention period for physician-maintained inpatient records [30]; however, this minimum may create a ‘liability trap’ as the Consumer Protection Act, 2019 establishes a 2-year limitation period from date of negligence discovery, which can extend significantly for latent injuries [49]. Consequently, best practices in medical defense litigation recommend retention periods of 5–10 years to align with potential civil liability exposure, even though the regulatory minimum is shorter. Indonesia’s 25-year requirement under Permenkes No. 24 of 2022 applies uniformly to all electronic medical records regardless of care modality, representing one of the longest retention mandates globally [31]. This requirement reflects a governmental policy emphasizing intergenerational care continuity and long-term digital health infrastructure investment, but imposes significant compliance burdens: data from Indonesian patients seen today must remain accessible, readable, and secure until 2050. Shorter retention periods may compromise long-term research and medicolegal protection, while extended requirements impose significant infrastructure burdens, particularly affecting low-resource settings [50].

### Specialty-driven standardization

Neurology-specific findings demonstrate the value of specialty-driven standardization. Professional societies like AHA/ASA, ESO, and WSO have developed detailed telestroke guidelines providing clear quality metrics and documentation standards [11, 15, 35]. These guidelines emphasize time-stamped documentation and standardized assessment scales as critical elements for quality telestroke care.

The emergence of platform regulation, exemplified by Singapore’s licensing model, represents a paradigm shift from individual practitioner accountability to systemic governance [40]. However, this approach may inadvertently create barriers for smaller providers and exacerbate digital divides between well-resourced and resource-limited settings.

### Liability framework implications

The concerning pattern of undefined liability frameworks in nearly one-third of jurisdictions creates uncertainty affecting specialist participation in teleconsultation networks. This regulatory vacuum produces a perverse inhibitory effect: in jurisdictions with ‘requesting physician primary liability’ models (31% of those with defined frameworks), generalist physicians in remote areas may hesitate to initiate specialist teleconsultations—particularly for high-acuity decisions such as thrombolysis—to avoid assuming sole medicolegal responsibility for treatment recommendations made by a third party [51]. This reluctance perpetuates healthcare access inequities in precisely the populations telemedicine was designed to serve. Concrete consequences of unclear liability frameworks include: (1) defensive documentation practices manifesting as exhaustive clinical reasoning documentation, extensive disclaimers about remote assessment limitations, and redundant consent processes that may increase consultation time [52, 53]; (2) specialist avoidance of high-acuity teleconsultation cases, with consultants limiting participation to low-risk follow-up encounters rather than acute decision-making scenarios [54]; (3) reduced access for complex acute conditions in resource-limited settings where specialist teleconsultation could provide greatest benefit but legal uncertainty creates disincentives [55]; and (4) insurance coverage gaps requiring supplementary coverage that may not be available or affordable for all practitioners [45].

While our analysis could not quantify precise impact on consultation volumes due to inconsistent data availability, qualitative evidence suggests medicolegal concerns serve as barriers to adoption [44, 45]. This has particular implications for equity, as specialist teleconsultation could extend expert care to underserved populations—a benefit undermined by legal uncertainty.

Critics of shared liability models argue they may increase administrative burden and documentation requirements without corresponding improvements in patient safety. However, proponents counter that explicit frameworks—regardless of model—provide clarity that facilitates rather than hinders teleconsultation adoption.

### Data governance and interoperability considerations

While this review focused primarily on documentation requirements, modern telemedicine documentation is inseparable from data interoperability standards. Long-term retention mandates—such as Indonesia’s 25-year requirement—presuppose not only data preservation but also sustained readability and portability across evolving technological platforms [56]. The HL7 FHIR (Fast Healthcare Interoperability Resources) standard has emerged as the predominant framework for health data exchange, enabling API-based integration across electronic health record systems, telemedicine platforms, and mobile health applications [57]. However, our analysis found that only 12 of 52 jurisdictions (23.1%) explicitly mandate interoperability standards in their telemedicine documentation regulations. This gap suggests that current regulatory frameworks may inadequately address the long-term accessibility of documented telemedicine encounters, potentially rendering meticulously retained records inaccessible due to format obsolescence. Future regulatory development should consider harmonizing documentation requirements with international data standards to ensure that retention mandates translate into meaningful long-term data availability.

### Equity and digital divide considerations

Our findings reveal concerning disparities in regulatory maturity correlating with economic development. High-income countries demonstrate comprehensive frameworks with detailed requirements, while low- and middle-income countries often lack resources for implementation and enforcement. This regulatory gap may paradoxically widen healthcare access disparities: telemedicine could democratize specialist access in resource-limited settings, yet inadequate regulatory frameworks may discourage international collaboration or investment in telemedicine infrastructure.

The heavy representation of high-income jurisdictions in our sample (Europe and Americas comprising 71.1% of analyzed jurisdictions) reflects both our search strategy limitations and genuine disparities in telemedicine regulatory development. Pacific Island nations, much of sub-Saharan Africa, and Central Asia remain underrepresented, limiting generalizability of our findings. Future research must prioritize these regions to understand unique regulatory challenges and opportunities.

Platform licensing requirements, while enhancing accountability in well-resourced settings, may create insurmountable barriers in low-resource contexts lacking technological infrastructure. Risk-based regulatory approaches matching requirements to clinical complexity could avoid over-regulating low-risk encounters while ensuring appropriate standards for complex care [58].

### Future research priorities

Comparative effectiveness studies examining different regulatory models’ impact on patient outcomes, access, and costs would provide empirical basis for policy decisions [59]. Implementation science research could identify factors distinguishing successful from struggling regulatory adoptions [60]. Quantitative analysis of the relationship between liability framework clarity and teleconsultation utilization requires standardized metrics across jurisdictions. Longitudinal studies tracking regulatory evolution post-pandemic will illuminate whether current trends toward permanent codification continue or revert.

### Practical recommendations and future perspectives

Synthesizing the findings of this scoping review, several practical recommendations emerge for the diverse stakeholders involved in telemedicine delivery. For regulators and policymakers, jurisdictions with undefined or ambiguous liability frameworks should prioritize the development of explicit medicolegal rules for tele-interconsultation, ideally through shared-liability models that have been shown in our analysis to be associated with the most comprehensive documentation requirements and the greatest legal clarity. Retention periods should be calibrated to align with statutes of limitations and the longitudinal needs of chronic disease care, while remaining technically feasible for low-resource settings. Risk-based regulatory approaches that match documentation requirements to clinical complexity should be favored over uniform prescriptive mandates, particularly when extending telemedicine to underserved populations.

For healthcare organizations and clinical leaders, the implementation of standardized telemedicine documentation templates aligned with applicable national regulations and specialty-specific guidelines (AHA/ASA, ESO, WSO) is essential, particularly for time-critical encounters such as telestroke. Organizations should embed structured fields capturing time-stamped clinical events, NIHSS components, contraindication assessment, and consent processes directly within electronic health record systems to reduce documentation burden and increase compliance. Cross-border programs require explicit interoperability planning using internationally recognized data standards such as HL7 FHIR, given that only 23.1% of jurisdictions analyzed currently mandate such standards.

For practicing neurologists and telestroke specialists, the heterogeneity of liability frameworks across jurisdictions requires explicit documentation of consultant qualifications, consultation rationale, and the integration of specialist recommendations into the patient’s care plan, particularly when operating across state or national borders. Clinicians should verify whether their professional liability insurance covers tele-interconsultation and cross-border encounters, given that 82.6% of surveyed insurers exclude international consultations. Training in remote neurological examination techniques, with explicit documentation of assessment limitations, should be incorporated into residency curricula and continuing medical education.

Looking forward, three trends are likely to shape the regulatory landscape over the next decade. First, we anticipate gradual convergence toward platform-based regulatory models exemplified by Singapore, balancing systemic accountability with operational flexibility, while specialty-driven standardization led by international professional societies will increasingly fill the gaps left by jurisdiction-specific regulation. Second, artificial-intelligence-enabled clinical decision support and automated documentation tools are expected to be progressively integrated into telemedicine workflows, raising new questions about liability allocation, algorithmic transparency, and documentation of AI-assisted decisions that current regulatory frameworks do not yet address. Third, the principle of mutual recognition—rather than full regulatory harmonization—will likely emerge as the most pragmatic pathway to enabling cross-border telemedicine while respecting legitimate national differences in legal traditions and healthcare system structures. Achieving these perspectives will require sustained collaboration among regulators, professional societies, healthcare organizations, technology developers, and patient representatives, supported by empirical evidence on the comparative effectiveness of different regulatory approaches in preserving patient safety, ensuring equitable access, and sustaining the long-term viability of telemedicine as a permanent component of modern healthcare delivery.

### Limitations

Several limitations warrant consideration. First, our search was primarily limited to English-language sources and those with available translations, potentially missing important non-Anglophone regulations despite translation efforts. The predominance of high-income jurisdictions (71.1%) in our sample limits generalizability to low- and middle-income settings. Second, rapid telemedicine regulatory evolution means some information may have been superseded by newer legislation between data collection and publication. Third, we examined regulations as written rather than implemented, unable to capture enforcement variations or practical interpretations. Fourth, heterogeneity of source types—ranging from binding legislation to professional guidelines—complicated direct comparisons. Fifth, our focus on documentation requirements may not fully capture the broader regulatory ecosystem influencing telemedicine practice. Finally, the regulatory intensity score, while innovative, requires external validation in diverse healthcare contexts to confirm weighting validity. Specifically, the score weighting inherently reflects value judgments about what constitutes regulatory maturity, and may systematically favor jurisdictions with prescriptive (civil law) regulatory traditions over those employing principles-based (common law) frameworks. Validation against objective outcomes such as patient safety metrics, malpractice claims incidence, and quality indicators would strengthen the score’s utility for cross-jurisdictional comparison [26, 27].

## Conclusion

Telemedicine has definitively transitioned from emergency contingency to regulated routine care globally, with the COVID-19 pandemic providing the evidence base for permanent regulatory frameworks that codify flexibility rather than revert to pre-pandemic restrictions. While core principles converge internationally—equivalent documentation standards, informed consent, and professional accountability—meaningful differences persist in implementation specifics, technology requirements, retention periods, and liability frameworks. The success of professional societies in developing detailed telestroke guidelines offers a model for specialty-driven requirements that can guide quality improvement. As healthcare becomes increasingly global and digital, the challenge is not eliminating regulatory diversity but managing it effectively through sophisticated compliance systems, international collaboration, and recognition that different regulatory approaches may achieve similar safety and quality objectives through different means. Finally, the goal should be interoperability and mutual recognition rather than uniformity, enabling telemedicine to fulfill its promise of democratizing access to quality healthcare while maintaining protective standards regardless of geography. This regulatory landscape provides a foundation for evidence-based policy development, informed clinical practice, and strategic organizational planning.

## Electronic Supplementary Material

Below is the link to the electronic supplementary material.

Supplementary Material 1 (DOCX 20.1 KB)

Supplementary Material 2 (DOCX 35.4 KB)

## Declarations

### General statement

All the authors have read and approved the submitted manuscript. The manuscript has not been submitted elsewhere nor published elsewhere in whole or in part.

### Generative AI and AI-assisted technologies in the writing process

During the preparation of this work the authors used CHAT GPT 5.1/5.2 and Claude Oppus 4.5 in order to review grammar and language. After using this tool/service, the authors reviewed and edited the content as needed and take full responsibility for the content of the publication. CHAT GPT 5.1/5.2 and Claude Oppus 4.5 were also employed to translate Portuguese into English.
