# Supplementary material for: Telemedicine documentation in neurology and telestroke: a global scoping review
Source: Neurol Sci. 2026 Jun 9;47(7):556. doi: 10.1007/s10072-026-09165-3 (PMC13246820; doi:10.1007/s10072-026-09165-3)
Supplement: Supplementary file 1 — Supplementary Material 1 (DOCX 20.1 KB) [file 10072_2026_9165_MOESM1_ESM.docx]

**Supplementary Material 1**

**Detailed Data by Jurisdiction**

**Table 1: Universal Documentation Requirements**

Patient identification (100% - 52/52 jurisdictions):

- Americas (19): USA, Canada, Brazil, Argentina, Colombia, Mexico, Peru, Chile, Uruguay, Paraguay, Venezuela, Ecuador, Bolivia, Costa Rica, Panama, Guatemala, Honduras, El Salvador, Nicaragua
- Europe (18): United Kingdom, Germany, France, Italy, Spain, Sweden, Denmark, Poland, Czech Republic, Hungary, Portugal, Romania, Netherlands, Belgium, Austria, Switzerland, Norway, Finland
- Asia-Pacific (12): Australia, Japan, Singapore, China, India, Malaysia, Indonesia, Thailand, Vietnam, New Zealand, South Korea, Philippines
- Africa (5): South Africa, Nigeria, Kenya, Ghana, Egypt
- Middle East (3): Dubai (UAE), Saudi Arabia, Israel

Two-factor verification (98% - 51/52):

- Exception: Ghana (accepts simple identification)
- Biometrics in Asia-Pacific (5/12 = 42%): China, India, Singapore, South Korea, Japan (15,18,21,24,28)

**Table 2: Medical Record Retention Periods**

3-5 years (8 jurisdictions):

- India (3 years), Thailand (5 years), Japan (5 years), Philippines (5 years), Vietnam (5 years initial), Israel (4 years), Egypt (5 years), Nicaragua (5 years) (15,21,35,38)

6-7 years (11 jurisdictions):

- Australia (7 years adults), United Kingdom (8 years adults), Canada (varies by province: Ontario 10, Quebec 5, BC 7), New Zealand (7 years), South Africa (6 years), Switzerland (7 years), Norway (7 years), Mexico (5 years), Guatemala (6 years), South Korea (7 years), Malaysia (7 years) (14,16,31,39)

8-10 years (14 jurisdictions):

- Germany (10 years), USA (varies by state: California 7, Texas 10, Florida 10, NY 8), Italy (10 years), Netherlands (10 years), Belgium (10 years), Austria (10 years), Denmark (10 years), Argentina (10 years), Uruguay (10 years), Paraguay (10 years), Dubai (10 years), Saudi Arabia (10 years), Singapore (8 years), Nigeria (10 years) (3,17,26,33,40)

11-15 years (9 jurisdictions):

- China (15 years), Colombia (15 years), Chile (15 years), Peru (15 years), Spain (15 years), Portugal (15 years), Finland (12 years), Hungary (15 years), Czech Republic (15 years) (18,28,29,37)

16-20 years (7 jurisdictions):

- France (20 years), Poland (20 years), Brazil (20 years), Sweden (20 years), Romania (20 years), Bolivia (20 years), Venezuela (20 years) (19,24,30,36)

20 years (3 jurisdictions):

- Kenya (20 years permanent), Indonesia (25 years), Vietnam (20 years for special cases) (20,35,38)

**Table 3: Telestroke - National Requirements**

Door-to-needle time documented (94% - 49/52):

- Not required: Ghana, Egypt, Nicaragua

Complete NIHSS (76% - 40/52):

- Required: All except 12 smaller African and Latin American jurisdictions (8,22,23)

Breakdown by requirement quality:

- Detailed requirements (n=26): USA, Canada, Germany, France, United Kingdom, Australia, Japan, Singapore, China, Brazil, Colombia, Argentina, Italy, Spain, Netherlands, Belgium, Switzerland, Austria, Denmark, Sweden, Norway, Finland, South Korea, Israel, Dubai, New Zealand (8,22,23,38)
- Basic requirements (n=26): Remaining jurisdictions

**Table S2: Platform Regulation Models**

Practitioner-only (23 jurisdictions - 44%):

- USA (most states), Canada (most provinces), Mexico, Chile, Uruguay, Paraguay, Venezuela, Ecuador, Bolivia, Peru, Costa Rica, Panama, Guatemala, Honduras, El Salvador, United Kingdom, Sweden, Denmark, Norway, Finland, Nigeria, Ghana, Egypt (3,16,31,39,41)

Platform certification (14 jurisdictions - 27%):

- Germany, France, Australia, Italy, Spain, Netherlands, Belgium, Austria, Switzerland, Portugal, Poland, Czech Republic, Hungary, Romania (14,17,24,36,44)

Platform licensing (8 jurisdictions - 15%):

- Dubai, Saudi Arabia, China, Indonesia, Thailand, Vietnam, Israel, Kenya (20,26,28,35,38,45)

**Integrated regulation (7 jurisdictions - 14%):**

**- Singapore, Malaysia, Brazil, India, Japan, South Korea, South Africa (12,24,25,39)**

**Table 4: Tele-interconsultation Liability**

Requesting physician primary liability (16 jurisdictions - 31%):

- China, Colombia, Chile, Peru, Argentina, Uruguay, Vietnam, Thailand, Indonesia, Malaysia, Philippines, Egypt, Ghana, Guatemala, Honduras, El Salvador (27,28,29,35,38)

Shared liability (12 jurisdictions - 23%):

- Brazil, Mexico, Venezuela, Ecuador, Bolivia, Paraguay, India, South Korea, Japan, Israel, Dubai, Saudi Arabia (15,21,26,30,32)

Consultant liability (8 jurisdictions - 15%):

- Canada (all provinces), Australia, New Zealand, United Kingdom, Germany, France, Italy, Spain (14,16,17,31,36)

Undefined/ambiguous (16 jurisdictions - 31%):

- USA (varies by state), Singapore, South Africa, Kenya, Nigeria, Poland, Czech Republic, Hungary, Romania, Portugal, Netherlands, Belgium, Austria, Switzerland, Denmark, Sweden, Norway, Finland, Costa Rica, Panama, Nicaragua (3,20,24,33,39,40,41)

**Additional References for Supplementary Material 2**

[S1] Ministry of Health Singapore. Telemedicine Platform Licensing Framework: Implementation Guidelines. Singapore: MOH; 2023.

[S2] Brazilian Federal Council of Medicine. Resolution CFM 2314/2022: Telemedicine Practice Standards. Brasília: CFM; 2022.

[S3] National Medical Commission India. Telemedicine Practice Guidelines. New Delhi: NMC; 2020.

[S4] South African Health Products Regulatory Authority. Guidance on Telemedicine Services. Pretoria: SAHPRA; 2021.
