# Supplementary material for: Telemedicine documentation in neurology and telestroke: a global scoping review
Source: Neurol Sci. 2026 Jun 9;47(7):556. doi: 10.1007/s10072-026-09165-3 (PMC13246820; doi:10.1007/s10072-026-09165-3)
Supplement: Supplementary file 2 — Supplementary Material 2 (DOCX 35.4 KB) [file 10072_2026_9165_MOESM2_ESM.docx]

**Supplementary Material 2**

**Table S1. Jurisdiction-by-Jurisdiction Matrix: Regulatory Sources for Documentation Requirements**

| **Jurisdiction** | **Patient Identification** | **Consent Documentation** | **Data Retention** | **Electronic Prescribing** | **Platform Regulation** | **Liability Framework** |
| --- | --- | --- | --- | --- | --- | --- |
| **AMERICAS** | | | | | | |
| United States | HIPAA 45 CFR §164.502 | State Medical Board Rules | State laws (varies 7-10 yrs) | DEA regulations, Ryan Haight Act | State licensing boards | State malpractice law |
| Canada | CMPA guidelines 2022 | Provincial College standards | Provincial laws (5-10 yrs) | Controlled Drugs Act | Provincial regulation | Consultant liable (CMPA) |
| Brazil | CFM Resolution 2314/2022 | CFM Resolution 2217/2018 | Law 13.787/2018 (20 years) | RDC 357/2020 ANVISA | Integrated (CFM + ANVISA) | Shared (CFM Res 2314) |
| Argentina | Law 25.326 (Personal Data) | Medical Ethics Code Art 49 | Resolution 1089/2012 (10 yrs) | ANMAT Disp 2318/2002 | Practitioner-only | Requesting physician primary |
| Colombia | Resolution 2654/2019 | Law 1581/2012 (Data) | Resolution 1995/1999 (15 yrs) | Decree 780/2016 | Practitioner-only | Requesting physician primary |
| Mexico | NOM-004-SSA3-2012 | LFPDPPP 2010 | NOM-004 (5 years) | Ley General de Salud | Practitioner-only | Shared liability |
| Chile | Law 20.584/2012 | Law 19.628 (Privacy) | Decree 570/2000 (15 yrs) | Decree 3/2010 | Practitioner-only | Requesting physician primary |
| Peru | RM 365-2020-MINSA | Law 29733/2011 (Data) | RM 214-2011 (15 years) | DS 014-2011-SA | Practitioner-only | Requesting physician primary |
| **EUROPE** | | | | | | |
| United Kingdom | GMC Good Practice 2023 | Mental Capacity Act 2005 | Records Management Code (8 yrs) | Human Medicines Reg 2012 | Practitioner-only (GMC) | Consultant liable |
| Germany | §630f BGB | GDPR Art 9 + §630d BGB | Berufsordnung (10 years) | E-Rezept mandatory 2024 | Platform certification (TI) | Consultant liable |
| France | Code Santé Publique L1111-7 | CSP R4127-36 | CSP R1112-7 (20 years) | Décret 2020-1545 | Platform certification (ARS) | Consultant liable |
| Italy | AGID Guidelines 2020 | Code Deontologia Art 33 | DPCM 178/2015 (10 years) | Law Decree 76/2020 | Platform certification | Consultant liable |
| Spain | RD 1720/2007 | Law 41/2002 Art 8 | Law 41/2002 (15 years) | RD 1302/2018 | Platform certification | Consultant liable |
| Netherlands | WGBO Art 454 | GDPR + WGBO | WGBO Art 454 (10 years) | Geneesmiddelenwet | Platform certification | Consultant liable |
| Belgium | Royal Decree 78/2017 | Law 22/08/2002 Art 8 | RD 78/2017 (10 years) | RD 21/01/2009 | Platform certification | Consultant liable |
| Poland | Act on Patient Rights 2008 | GDPR + Act 2008 | Act 2008 (20 years) | E-prescribing Act 2020 | Platform certification | Consultant liable |
| Sweden | Patient Data Act 2008:355 | GDPR + Consent Act | PDA 2008 (20 years) | Medicinal Products Act | Practitioner-only | Consultant liable |
| **ASIA-PACIFIC** | | | | | | |
| Australia | Privacy Act 1988 | Medical Board Guidelines | State laws (7 years) | PBS Online | Practitioner-only (AHPRA) | Consultant liable |
| Japan | Medical Care Act Art 24 | Personal Info Protection Act | Medical Care Act (5 years) | Pharmaceutical Act | Integrated regulation | Shared liability |
| Singapore | HCSA 2020 | PDPA 2012 + HCSA | Private Hospitals Reg (10 yrs) | Medicines Act Ch 176 | Platform licensing (MOH) | Integrated framework |
| China | Cybersecurity Law 2017 | Telemedicine Measures 2018 | Hospital Management Reg (15 yrs) | Drug Admin Law 2019 | Platform licensing (NHSA) | Requesting physician primary |
| India | NMC Regulations 2023 | Digital Information Security Act | NMC Reg Ch 3 (3 years) | Drugs & Cosmetics Act | Integrated (NMC + Ayushman) | Shared liability |
| South Korea | Medical Service Act Art 22 | PIPA 2011 | Medical Service Act (7 yrs) | Pharmaceutical Affairs Act | Integrated regulation | Shared liability |
| Indonesia | Permenkes 20/2019 | UU 36/2009 (Health) | Permenkes 24/2022 (25 yrs) | Permenkes 3/2020 | Platform licensing | Requesting physician primary |
| Malaysia | Telemedicine Act 1997 | Medical Act 1971 | Private Healthcare Reg (7 yrs) | Poison Act 1952 | Integrated (MMC) | Requesting physician primary |
| Thailand | Ministerial Reg 2020 | PDPA 2019 | Medical Registration Act (5 yrs) | Drug Act BE 2510 | Platform licensing | Requesting physician primary |
| New Zealand | Privacy Act 2020 | Code of Health Conduct | Health Info Privacy Code (7 yrs) | Medicines Act 1981 | Practitioner-only (MCNZ) | Consultant liable |
| **MIDDLE EAST & AFRICA** | | | | | | |
| Dubai (UAE) | DHA Standard TP-38 | Federal Law 2/2019 | DHA Standards (10 years) | MOHAP Resolution 2019 | Platform licensing (DHA) | Shared liability |
| Saudi Arabia | CCHI Policy 2020 | Data Protection Law 2021 | MOH Circular (10 years) | SFDA Guidelines | Platform licensing (NPHIES) | Shared liability |
| Israel | Patient Rights Law 1996 | Privacy Protection Law | MOH Directive (4 years) | Pharmacy Ordinance | Platform licensing | Shared liability |
| South Africa | POPIA 2013 | NHA 2003 Ch 2 | NHA Regulations (6 years) | Medicines Act 101/1965 | Integrated (HPCSA) | Consultant liable |
| Kenya | Data Protection Act 2019 | Health Act 2017 | MOH Standards (20 years) | Pharmacy Act 2012 | Platform licensing | Undefined framework |
| Egypt | Law 151/2019 (Health) | Personal Data Protection Law | MOH Decree (5 years) | Pharmacy Law 127/1955 | Practitioner-only | Requesting physician primary |

**Legend**. This table maps specific regulatory sources (legislation, regulations, guidelines, and policies) to each core documentation requirement across 52 jurisdictions. Regulatory sources include binding legislation, government regulations, professional regulatory body requirements, and authoritative clinical practice guidelines. Jurisdictions are organized by geographic region. Abbreviations: HIPAA = Health Insurance Portability and Accountability Act; CFM = Conselho Federal de Medicina; GDPR = General Data Protection Regulation; GMC = General Medical Council; HCSA = Healthcare Services Act; CMPA = Canadian Medical Protective Association; NMC = National Medical Commission; DHA = Dubai Health Authority; POPIA = Protection of Personal Information Act; MOH = Ministry of Health.

**Table S2: Documentation Requirements for Chronic Neurological Conditions**

| Condition | Specific Scales Required | Frequency Tracking | Medication Response | Quality of Life | Caregiver Assessment |
| --- | --- | --- | --- | --- | --- |
| Epilepsy | ILAE classification (67%) | Seizure diary (89%) | Side effects (91%) | Optional (34%) | When applicable (56%) |
| Parkinson's | Modified UPDRS (71%) | Motor fluctuations (78%) | On/off periods (83%) | Recommended (67%) | Often required (61%) |
| Dementia | Cognitive scale (93%) | Decline rate (76%) | Behavioral response (81%) | Required (71%) | Usually required (89%) |
| Multiple Sclerosis | EDSS or variant (64%) | Relapse log (91%) | Treatment response (87%) | Recommended (58%) | When applicable (43%) |
| Headache | Headache diary (73%) | Trigger identification (67%) | Medication use (94%) | Impact scales (61%) | Rarely required (12%) |

**Legend**. Specialty-specific documentation requirements for five major neurological conditions across 43 jurisdictions with neurology-specific regulations. ILAE = International League Against Epilepsy; UPDRS = Unified Parkinson's Disease Rating Scale; EDSS = Expanded Disability Status Scale. Dementia showed the highest documentation standardization with cognitive scales required in 93% of jurisdictions, while headache disorders had the least caregiver documentation requirements (12%), reflecting differences in disease burden on care partners. Quality of life metrics were most frequently mandated for dementia (71%) and Parkinson's disease (67%).

**Table S3: Technology Platform Regulatory Models**

| Model | Description | Jurisdictions n (%) | Key Features | Examples |
| --- | --- | --- | --- | --- |
| Practitioner-only | Platform unregulated | 23 (44%) | Provider liability only | USA states, Canada |
| Platform certification | Technical standards | 14 (27%) | Security/quality audits | Germany, France, Australia |
| Platform licensing | Operating license required | 8 (15%) | Facility-level regulation | Dubai, Saudi Arabia, China |
| Integrated regulation | Joint accountability | 7 (14%) | Shared liability model | Singapore, Malaysia, Brazil |

**Legend**. Distribution of regulatory approaches to telemedicine technology platforms across 52 jurisdictions. Platform regulation showed significant correlation with healthcare system structure (χ² = 12.34, p < 0.05). The practitioner-only model dominated in federated systems (USA, Canada), while integrated regulation appeared primarily in countries with unified national health systems. Middle Eastern countries demonstrated the highest adoption of platform licensing (Dubai 88%, Saudi Arabia 85%), requiring facility-level accountability beyond individual practitioner responsibility.

**Table S4: Primary Implementation Barriers by Region**

| Barrier | Americas | Europe | Asia-Pacific | Africa/ME | Overall Rank |
| --- | --- | --- | --- | --- | --- |
| Cross-jurisdictional licensing | 94% | 67% | 58% | 42% | 1 |
| Technology infrastructure | 31% | 28% | 47% | 89% | 2 |
| Provider awareness/training | 63% | 44% | 61% | 78% | 3 |
| EHR integration | 58% | 61% | 53% | 67% | 4 |
| Reimbursement alignment | 78% | 39% | 42% | 56% | 5 |
| Patient digital literacy | 42% | 47% | 58% | 81% | 6 |

**Legend**. Percentage of regulatory sources identifying specific barriers to telemedicine documentation compliance, stratified by region. Cross-jurisdictional licensing emerged as the dominant barrier globally (73%), but showed marked regional variation from 94% in the Americas to 42% in Africa/Middle East. Infrastructure limitations showed inverse patterns, representing the primary barrier in Africa/ME (89%) versus minimal concern in Europe (28%). These disparities reflect fundamental differences between high-income countries focusing on regulatory integration versus low-middle-income countries addressing basic access enablement.

# **Documentary Sources and Regulatory References**

## **AMERICAS**

**United States**

1. [HIPAA Privacy Rule 45 CFR §164.502](https://www.ecfr.gov/current/title-45/subtitle-A/subchapter-C/part-164)

2. [DEA Telemedicine Regulations](https://www.deadiversion.usdoj.gov/pubs/manuals/pract/section5.htm)

3. [Ryan Haight Online Pharmacy Consumer Protection Act](https://www.deadiversion.usdoj.gov/schedules/orangebook/e_cs_sched.pdf)

4. [Federation of State Medical Boards - Telemedicine Guidelines](https://www.fsmb.org/siteassets/advocacy/policies/telemedicine-policies.pdf)

**Canada**

1. [CMPA Good Practices Guide - Telemedicine](https://www.cmpa-acpm.ca/en/advice-publications/browse-articles/2022/telemedicine-privacy-and-security)

2. [College of Physicians and Surgeons of Ontario - Telemedicine](https://www.cpso.on.ca/Physicians/Policies-Guidance/Policies/Telemedicine)

3. [Controlled Drugs and Substances Act](https://laws-lois.justice.gc.ca/eng/acts/c-38.8/)

**Brazil**

1. [CFM Resolution 2314/2022 - Telemedicine](https://sistemas.cfm.org.br/normas/visualizar/resolucoes/BR/2022/2314)

2. [CFM Resolution 2217/2018 - Medical Ethics Code](https://portal.cfm.org.br/images/PDF/cem2019.pdf)

3. [Law 13.787/2018 - Digital Health Records](http://www.planalto.gov.br/ccivil_03/_ato2015-2018/2018/lei/L13787.htm)

4. [ANVISA RDC 357/2020 - Electronic Prescribing](https://www.in.gov.br/en/web/dou/-/resolucao-rdc-n-357-de-24-de-marco-de-2020-250002051)

**Argentina**

1. [Law 25.326 - Personal Data Protection](http://servicios.infoleg.gob.ar/infolegInternet/anexos/60000-64999/64790/norma.htm)

2. [Resolution 1089/2012 - Medical Records](https://www.argentina.gob.ar/normativa/nacional/resoluci%C3%B3n-1089-2012-197866)

3. [ANMAT Disposition 2318/2002](http://www.anmat.gov.ar/webanmat/legislacion/medicamentos/Disposicion_ANMAT_2318-2002.pdf)

**Colombia**

1. [Resolution 2654/2019 - Telemedicine Standards](https://www.minsalud.gov.co/Normatividad_Nuevo/Resoluci%C3%B3n%20No.%202654%20de%202019.pdf)

2. [Law 1581/2012 - Personal Data Protection](https://www.sic.gov.co/sites/default/files/documentos/LEY_1581_2012.pdf)

3. [Resolution 1995/1999 - Medical Records](https://www.minsalud.gov.co/Normatividad_Nuevo/RESOLUCI%C3%93N%201995%20DE%201999.pdf)

4. [Decree 780/2016 - Health Sector Regulation](https://www.minsalud.gov.co/Normatividad_Nuevo/Decreto%200780%20de%202016.pdf)

**Mexico**

1. [NOM-004-SSA3-2012 - Medical Records](https://www.dof.gob.mx/nota_detalle.php?codigo=5272787&fecha=15/10/2012)

2. [Federal Law on Protection of Personal Data (LFPDPPP)](http://www.diputados.gob.mx/LeyesBiblio/pdf/LFPDPPP.pdf)

3. [General Health Law](http://www.diputados.gob.mx/LeyesBiblio/pdf/142_190221.pdf)

**Chile**

1. [Law 20.584/2012 - Patient Rights](https://www.bcn.cl/leychile/navegar?idNorma=1039348)

2. [Law 19.628 - Protection of Privacy](https://www.bcn.cl/leychile/navegar?idNorma=141599)

3. [Decree 570/2000 - Medical Records Regulation](https://www.bcn.cl/leychile/navegar?idNorma=181979)

**Peru**

1. [RM 365-2020-MINSA - Telemedicine Technical Document](https://cdn.www.gob.pe/uploads/document/file/866328/RM_365-2020-MINSA.PDF)

2. [Law 29733/2011 - Personal Data Protection](https://www.minjus.gob.pe/wp-content/uploads/2014/03/LEY-29733.pdf)

3. [RM 214-2011/MINSA - Medical Records](http://bvs.minsa.gob.pe/local/MINSA/1782.pdf)

## **EUROPE**

**United Kingdom**

1. [GMC Good Medical Practice 2024](https://www.gmc-uk.org/ethical-guidance/ethical-guidance-for-doctors/good-medical-practice)

2. [Mental Capacity Act 2005](https://www.legislation.gov.uk/ukpga/2005/9/contents)

3. [Records Management Code of Practice](https://www.gov.uk/government/publications/records-management-code-of-practice-for-health-and-social-care)

4. [Human Medicines Regulations 2012](https://www.legislation.gov.uk/uksi/2012/1916/contents)

**Germany**

1. [§630f BGB - Medical Documentation](https://www.gesetze-im-internet.de/bgb/__630f.html)

2. [GDPR Article 9 - Processing Special Categories](https://gdpr-info.eu/art-9-gdpr/)

3. [Berufsordnung für Ärzte](https://www.bundesaerztekammer.de/fileadmin/user_upload/BAEK/Themen/Internationales/Berufsordnung_EN.pdf)

4. [E-Rezept Information](https://www.kbv.de/html/e-rezept.php)

**France**

1. [Code de la Santé Publique L1111-7](https://www.legifrance.gouv.fr/codes/article_lc/LEGIARTI000006685767)

2. [Code de déontologie médicale R4127-36](https://www.legifrance.gouv.fr/codes/article_lc/LEGIARTI000006913000)

3. [Décret 2020-1545 - Electronic Prescriptions](https://www.legifrance.gouv.fr/jorf/id/JORFTEXT000042665334)

**Italy**

1. [AGID Guidelines 2020 - Telemedicine](https://www.agid.gov.it/it/agenzia/stampa-e-comunicazione/notizie)

2. [Code of Medical Ethics Article 33](https://portale.fnomceo.it/codice-deontologico/)

3. [DPCM 178/2015 - Electronic Medical Records](https://www.gazzettaufficiale.it/eli/id/2015/11/11/15A08202/sg)

**Spain**

1. [Royal Decree 1720/2007 - Electronic Health Records](https://www.boe.es/buscar/act.php?id=BOE-A-2008-979)

2. [Law 41/2002 - Patient Autonomy](https://www.boe.es/buscar/act.php?id=BOE-A-2002-22188)

3. [Royal Decree 1302/2018 - Electronic Prescription](https://www.boe.es/buscar/act.php?id=BOE-A-2018-14719)

**Netherlands**

1. [WGBO Article 454 - Medical Treatment Agreement](https://wetten.overheid.nl/BWBR0005290/2022-07-01#Boek7_Titeldeel7_Afdeling5)

2. [GDPR Implementation](https://autoriteitpersoonsgegevens.nl/en/about-ap/about-the-dutch-dpa)

3. [Geneesmiddelenwet - Medicines Act](https://wetten.overheid.nl/BWBR0021505/2023-01-01)

**Belgium**

1. [Royal Decree 78/2017 - Electronic Health Records](https://www.ejustice.just.fgov.be/cgi_loi/change_lg.pl?language=nl&la=N&cn=2017111009&table_name=wet)

2. [Law 22/08/2002 - Patient Rights](https://www.ejustice.just.fgov.be/cgi_loi/change_lg.pl?language=fr&la=F&cn=2002082245&table_name=loi)

3. [E-Prescription Royal Decree](https://www.recip-e.be/)

**Poland**

1. [Act on Patient Rights 2008](https://isap.sejm.gov.pl/isap.nsf/DocDetails.xsp?id=wdu20081640111)

2. [E-Prescription Act 2020](https://www.gov.pl/web/zdrowie/e-recepta)

3. [GDPR Implementation Poland](https://uodo.gov.pl/en)

**Sweden**

1. [Patient Data Act 2008:355](https://www.riksdagen.se/sv/dokument-lagar/dokument/svensk-forfattningssamling/patientdatalag-2008355_sfs-2008-355)

2. [Medicinal Products Act](https://www.lakemedelsverket.se/en/regulation/laws)

3. [E-Health Authority](https://www.ehalsomyndigheten.se/)

## **ASIA-PACIFIC**

**Australia**

1. [Privacy Act 1988](https://www.legislation.gov.au/Details/C2021C00452)

2. [Medical Board of Australia - Telehealth Guidelines](https://www.medicalboard.gov.au/Codes-Guidelines-Policies/Telehealth-guidance.aspx)

3. [PBS Online](https://www.servicesaustralia.gov.au/prescription-shopping-program)

**Japan**

1. [Medical Care Act Article 24](https://www.japaneselawtranslation.go.jp/en/laws/view/3896)

2. [Personal Information Protection Act](https://www.ppc.go.jp/en/legal/)

3. [Pharmaceutical Affairs Act](https://www.japaneselawtranslation.go.jp/en/laws/view/3712)

**Singapore**

1. [Healthcare Services Act 2020](https://sso.agc.gov.sg/Act/HCSA2020)

2. [Personal Data Protection Act 2012](https://sso.agc.gov.sg/Act/PDPA2012)

3. [MOH Licensing Requirements](https://www.moh.gov.sg/licensing-and-regulation/regulations-guidelines-and-circulars)

4. [Medicines Act Chapter 176](https://sso.agc.gov.sg/Act/MA1975)

**China**

1. [Cybersecurity Law 2017](http://www.npc.gov.cn/englishnpc/c23934/202112/1abd88c73e5f4464a4660c3b76a4fa76.shtml)

2. [Internet Diagnosis and Treatment Measures 2018](http://www.nhc.gov.cn/yzygj/s3594/201809/e3ad9ed4e32d4cdea4fee1b76a3d9b5f.shtml)

3. [Drug Administration Law 2019](http://www.npc.gov.cn/englishnpc/c23934/202001/49d0a3db2fc74c75a5a290a77ae0ede8.shtml)

**India**

1. [NMC Professional Conduct Regulations 2023](https://www.nmc.org.in/rules-regulations/code-of-ethics/)

2. [Digital Information Security in Healthcare Act Draft](https://www.mohfw.gov.in/)

3. [Drugs and Cosmetics Act 1940](https://cdsco.gov.in/opencms/opencms/en/Acts-Rules/Drugs-Cosmetics-Act-Rules/)

**South Korea**

1. [Medical Service Act Article 22](https://elaw.klri.re.kr/eng_service/lawView.do?hseq=53530&lang=ENG)

2. [Personal Information Protection Act 2011](https://www.pipc.go.kr/np/eng/main.do)

3. [Pharmaceutical Affairs Act](https://elaw.klri.re.kr/eng_service/lawView.do?hseq=55839&lang=ENG)

**Indonesia**

1. [Permenkes 20/2019 - Telemedicine Services](https://peraturan.bpk.go.id/Home/Details/140482/permenkes-no-20-tahun-2019)

2. [UU 36/2009 - Health](https://peraturan.bpk.go.id/Home/Details/38778/uu-no-36-tahun-2009)

3. [Permenkes 24/2022 - Medical Records](https://peraturan.bpk.go.id/Home/Details/206459/permenkes-no-24-tahun-2022)

4. [Permenkes 3/2020 - Hospital Classification](https://peraturan.bpk.go.id/Home/Details/131760/permenkes-no-3-tahun-2020)

**Malaysia**

1. [Telemedicine Act 1997](https://www.moh.gov.my/moh/resources/Penerbitan/Garis%20Panduan/Telemedicine/TELEMEDICINE_ACT_1997.pdf)

2. [Medical Act 1971](https://www.mmc.gov.my/wp-content/uploads/2019/06/Medical-Act-1971.pdf)

3. [Private Healthcare Facilities Regulations](https://www.moh.gov.my/moh/resources/Penerbitan/Garis%20Panduan/Umum_(Awam)/PRIVATE_HEALTHCARE_FACILITIES_ACT_1998.pdf)

**Thailand**

1. [Ministerial Regulation on Telemedicine 2020](http://www.ratchakitcha.soc.go.th/DATA/PDF/2563/E/127/T_0001.PDF)

2. [Personal Data Protection Act 2019](https://www.pdpc.or.th/pdpaen.html)

3. [Drug Act B.E. 2510](https://www.fda.moph.go.th/sites/drug/en/SitePages/Home.aspx)

**New Zealand**

1. [Privacy Act 2020](https://www.legislation.govt.nz/act/public/2020/0031/latest/LMS23223.html)

2. [Medical Council Code of Ethics](https://www.mcnz.org.nz/our-standards/current-standards/statement-on-telehealth/)

3. [Health Information Privacy Code](https://www.privacy.org.nz/privacy-act-2020/codes-of-practice/hipc2020/)

4. [Medicines Act 1981](https://www.legislation.govt.nz/act/public/1981/0118/latest/DLM53790.html)

## **MIDDLE EAST & AFRICA**

**Dubai (UAE)**

1. [DHA Standard TP-38 - Telemedicine](https://www.dha.gov.ae/en/HealthRegulation/Pages/TelehealthStandards.aspx)

2. [Federal Law 2/2019 - Use of ICT in Health](https://u.ae/en/about-the-uae/strategies-initiatives-and-awards/federal-governments-strategies-and-plans/digital-health)

3. [MOHAP Resolutions](https://www.mohap.gov.ae/en/knowledge-center/laws-regulations)

**Saudi Arabia**

1. [CCHI Telemedicine Policy 2020](https://www.cchi.gov.sa/en/Regulations/Documents/Regulations%20&%20Guidelines.pdf)

2. [Personal Data Protection Law 2021](https://sdaia.gov.sa/en/PDPL/Pages/default.aspx)

3. [SFDA Guidelines](https://www.sfda.gov.sa/en)

4. [NPHIES Platform](https://nphies.sa/)

**Israel**

1. [Patient Rights Law 1996](https://www.health.gov.il/English/Topics/RightsInsured/Rights_of_patients/Pages/default.aspx)

2. [Privacy Protection Law 1981](https://www.gov.il/en/departments/guides/privacy-protection-law)

3. [Ministry of Health Directives](https://www.health.gov.il/English/Pages/HomePage.aspx)

**South Africa**

1. [POPIA 2013 - Protection of Personal Information](https://popia.co.za/)

2. [National Health Act 2003](https://www.gov.za/documents/national-health-act)

3. [Medicines and Related Substances Act 101/1965](https://www.sahpra.org.za/documents/medicines-act/)

4. [HPCSA Guidelines](https://www.hpcsa.co.za/Publications/Ethics)

**Kenya**

1. [Data Protection Act 2019](http://kenyalaw.org/kl/fileadmin/pdfdownloads/Acts/2019/TheDataProtectionAct__No24of2019.pdf)

2. [Health Act 2017](http://kenyalaw.org/kl/fileadmin/pdfdownloads/Acts/2017/TheHealthActNo.21of2017.pdf)

3. [Pharmacy and Poisons Act 2012](http://kenyalaw.org/kl/fileadmin/pdfdownloads/Acts/PharmacyandPoisonsAct_Cap244.pdf)

**Egypt**

1. [Law 151/2019 - Universal Health Insurance](https://www.ilo.org/dyn/natlex/natlex4.detail?p_lang=en&p_isn=111134)

2. [Personal Data Protection Law](https://www.dataguidance.com/notes/egypt-data-protection-overview)

3. [Ministry of Health Regulations](https://www.mohp.gov.eg/Default.aspx)
